# Low PD-L1 Expression Strongly Correlates with Local Recurrence in Epstein-Barr Virus-Positive Nasopharyngeal Carcinoma after Radiation-Based Therapy

**DOI:** 10.3390/cancers10100374

**Published:** 2018-10-09

**Authors:** Yu-Jen Liu, Ngan-Ming Tsang, Chuen Hsueh, Chi-Ju Yeh, Shir-Hwa Ueng, Tong-Hong Wang, Wen-Yu Chuang

**Affiliations:** 1 Department of Pathology, Chang Gung Memorial Hospital and Chang Gung University, 5 Fusing Street, Gueishan, Taoyuan 333, Taiwan; yjl777@cgmh.org.tw (Y.-J.L.); ch9211@adm.cgmh.org.tw (C.H.); taki.yeh@gmail.com (C.-J.Y.); susie.ueng@gmail.com (S.-H.U.); 2 Department of Radiation Oncology, Chang Gung Memorial Hospital and Chang Gung University, 5 Fusing Street, Gueishan, Taoyuan 333, Taiwan; vstsang@cgmh.org.tw; 3 Chang Gung Molecular Medicine Research Center, Chang Gung University, 259 Wenhua First Road, Gueishan, Taoyuan 333, Taiwan; 4 Tissue Bank, Chang Gung Memorial Hospital, 5 Fusing Street, Gueishan, Taoyuan 333, Taiwan; cellww@adm.cgmh.org.tw; 5 Center for Vascularized Composite Allotransplantation, Chang Gung Memorial Hospital, 5 Fusing Street, Gueishan, Taoyuan 333, Taiwan

**Keywords:** programmed death-ligand 1, nasopharyngeal carcinoma, Epstein-Barr virus, immunohistochemistry, radiotherapy, local recurrence, survival

## Abstract

The prognostic value of programmed death-ligand 1 (PD-L1) expression in nasopharyngeal carcinoma (NPC) is controversial, with previous studies showing conflicting results. Most NPCs in endemic areas are Epstein-Barr virus (EBV)-positive. Our aim was to evaluate the clinical significance of PD-L1 expression in EBV-positive NPC. We retrospectively analyzed PD-L1 expression on tumor cells (TCs) and immune cells (ICs) by immunohistochemistry in 208 EBV-positive NPC patients who underwent radiotherapy (203 with concurrent chemotherapy). The percentages of TCs and ICs expressing PD-L1 were evaluated respectively. There was a strong correlation between local recurrence and low PD-L1 expression on ICs (*p* = 0.0012), TCs (*p* = 0.013) or both (*p* = 0.000044), whereas all clinical parameters had no influence on local recurrence. Using multivariate analysis, low PD-L1 expression on ICs was an independent adverse prognostic factor (*p* = 0.0080; HR = 1.88; 95% CI = 1.18–3.00) for disease-free survival. High PD-L1 expression on both ICs and TCs was an independent favorable prognostic factor (*p* = 0.022; HR = 0.46; 95% CI = 0.24–0.89) for overall survival. We show for the first time that low PD-L1 expression on ICs and TCs strongly correlates with local recurrence in EBV-positive NPC patients after radiation-based therapy. A simple immunohistochemical study for PD-L1 can identify patients prone to local recurrence, and such patients might benefit from more aggressive treatment in future clinical trials.

## 1. Introduction

Nasopharyngeal carcinoma (NPC) is a malignancy with distinct etiology, histopathology and geographic distribution. There are approximately 87,000 new cases per year in the world [1]. NPC is uncommon among Caucasians, with an age-adjusted annual incidence of less than 1 case per 100,000 persons [2]. The incidence is disproportionally high in some ethnic groups, including the Inuit, northern Africans, and Chinese from south-eastern Asia [2]. The age-standardized incidence of NPC in 2012 was 6.96 cases and 16.5 cases per 100,000 in Taiwan [3] and Hong Kong [2], respectively.

Currently, therapeutic decisions for NPC are mainly based on tumor stage [4]. Early stage disease can be treated with radiotherapy alone, whereas the more advanced disease is treated with concurrent chemoradiotherapy. In recent decades, the prognosis of NPC has improved significantly due to advances in diagnostic imaging, radiotherapy technology, and the wider use of systemic therapy [4]. However, even with the best available treatment, about 5–15% of patients later develop local recurrence, and about 15–30% of patients finally experience failure at distant sites [4]. Despite some successful salvage therapy in highly selected patients with local recurrence, most NPC patients with recurrent disease can only be treated with palliative chemotherapy [4]. A method to predict recurrence, if available, would be invaluable for developing a better therapeutic strategy in the future.

In the 2017 World Health Organization (WHO) classification of head and neck tumors [2], NPC is divided into three major histologic subtypes: keratinizing squamous cell carcinoma (SCC), non-keratinizing SCC, and basaloid SCC. In the endemic areas of NPC, including Taiwan, most cases are of the non-keratinizing subtype. The strong association between Epstein-Barr virus (EBV) infection and non-keratinizing NPC has been well established [5,6].

The EBV-positive NPC is associated with dense infiltrates of lymphocytes. Most intermixed lymphocytes in NPC are T cells, and cytotoxic tumor-infiltrating T lymphocytes have been reported to be a favorable prognostic factor in NPC patients [7,8]. Recently, immunotherapy targeting immune checkpoints, such as cytotoxic T-lymphocyte-associated protein 4 (CTLA-4), programmed cell death-1 (PD-1), programmed death-ligand 1 (PD-L1), and lymphocyte activation gene 3 (LAG3), has become one of the promising treatment modalities in a variety of malignancies [9,10,11,12]. Since the immune checkpoint inhibitors activate cytotoxic T cells to attack cancer cells, patients with lymphocyte-rich cancer types (such as EBV-positive NPC) might benefit more from such immunotherapy.

Immunotherapeutic agents blocking the PD-1/PD-L1 signaling axis have achieved remarkable treatment effects in patients with various types of cancers, including EBV-associated malignancies [13,14]. It is also known that the PD-L1 expression level in the tumor may be of predictive value for treatment efficacy in some cancer types [15,16,17]. Recently, a monoclonal anti-PD-1 antibody pembrolizumab has been shown to exhibit anti-tumor effects in patients with PD-L1-positive NPC [18]. However, the clinical significance of PD-L1 expression in NPC remains controversial, with previous studies showing conflicting conclusions [8,19,20,21,22,23,24,25,26,27,28]. In the present study, we evaluated the clinical significance of PD-L1 expression on tumor cells (TCs) and immune cells (ICs) in 208 NPC patients who underwent radiation-based therapy.

## 2. Results

### 2.1. PD-L1 Expression and Clinicopathologic Characteristics

**Figure 1:**
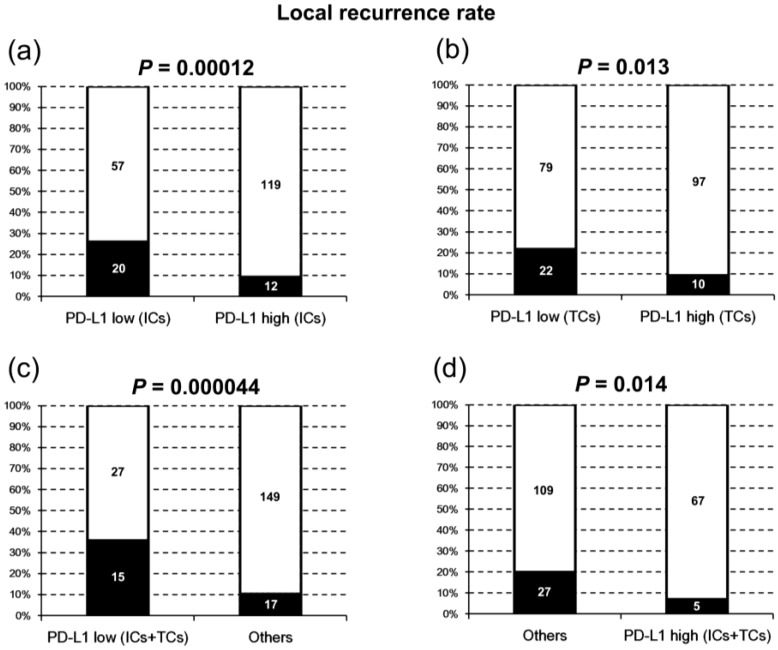
The local recurrence rates in patients with different levels of programmed death-ligand 1 (PD-L1) expression on immune cells (ICs) and tumor cells (TCs).

In our cases, the median percentage of PD-L1-positive cells was 4% and 2% on TCs and ICs, respectively. For TCs, a percentage of positive cells lower than 4% was considered low expression (otherwise high expression). For ICs, a percentage of positive cells lower than 2% was considered low expression (otherwise high expression). There was no significant correlation of PD-L1 expression level between TCs and ICs (*p* = 0.185). The clinicopathologic characteristics of our patients grouped by PD-L1 expression on TCs or ICs are listed in Table 1. Of note, local recurrence was strongly associated with low PD-L1 expression on ICs (*p* = 0.0012), TCs (*p* = 0.013) or both (*p* = 0.000044) (Table 1; Figure 1), whereas all clinical parameters had no significant influence on local recurrence. PD-L1 expression had no influence on other clinical characteristics, including neck recurrence and distant metastasis (Table 1).

### 2.2. PD-L1 Expression and Local Recurrence-Free Survival

**Figure 2:**
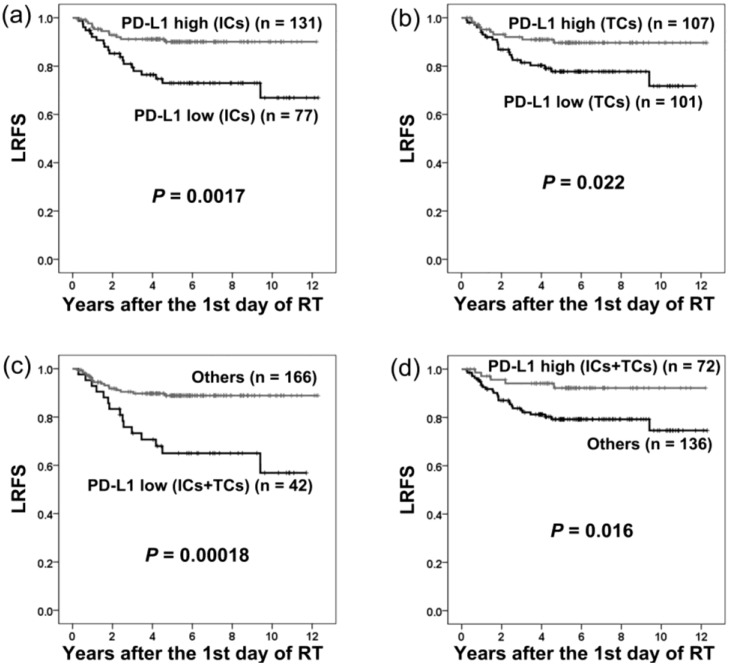
Local recurrence-free survival (LRFS) in patients with different levels of PD-L1 expression on immune cells (ICs) and tumor cells (TCs). There was significantly shorter LRFS in patients with low PD-L1 expression on ICs (**a**), TCs (**b**), or both ICs and TCs (**c**). Significantly longer LRFS was observed in patients with high PD-L1 expression on both ICs and TCs (**d**).

The local recurrence-free survival (LRFS) of patients grouped by PD-L1 expression is shown in Figure 2. LRFS was significantly shorter in patients with low PD-L1 expression on ICs (*p* = 0.0017), TCs (*p* = 0.022), or both (*p* = 0.00018; Figure 2).

Using univariate analysis, low PD-L1 expression on ICs (*p* = 0.0028; HR = 2.99; 95% CI = 1.46–6.12), TCs (*p* = 0.026; HR = 2.34; 95% CI = 1.11–4.95), or both (*p* = 0.00045; HR = 3.48; 95% CI = 1.74–6.98; Table 2) correlated with adverse prognosis for LRFS. All clinical parameters had no significant influence on LRFS (Table 2).

Using multivariate analysis, low PD-L1 expression on ICs was the only independent adverse prognostic factor for LRFS (*p* = 0.0062; HR = 2.74; 95% CI = 1.33–5.63; Table 2).

### 2.3. PD-L1 Expression and Distant Metastasis-Free Survival

**Figure 3:**
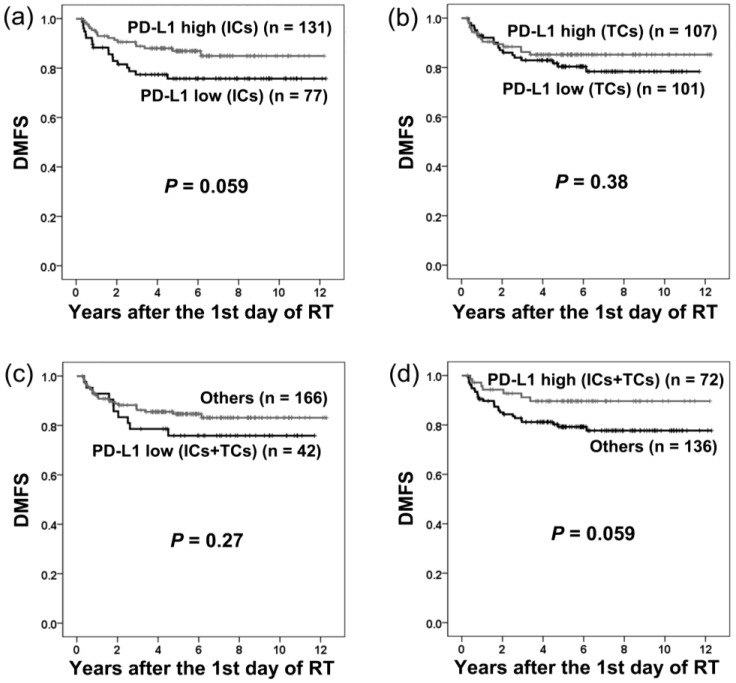
Distant metastasis-free survival (DMFS) in patients with different levels of PD-L1 expression on immune cells (ICs) and tumor cells (TCs). PD-L1 expression on ICs (**a**), TCs (**b**), or both ICs and TCs (**c**,**d**) had no significant influence on DMFS.

The distant metastasis-free survival (DMFS) of patients grouped by PD-L1 expression is shown in Figure 3. PD-L1 expression in either ICs or TCs had no significant influence on DMFS.

### 2.4. PD-L1 Expression and Disease-free Survival

**Figure 4:**
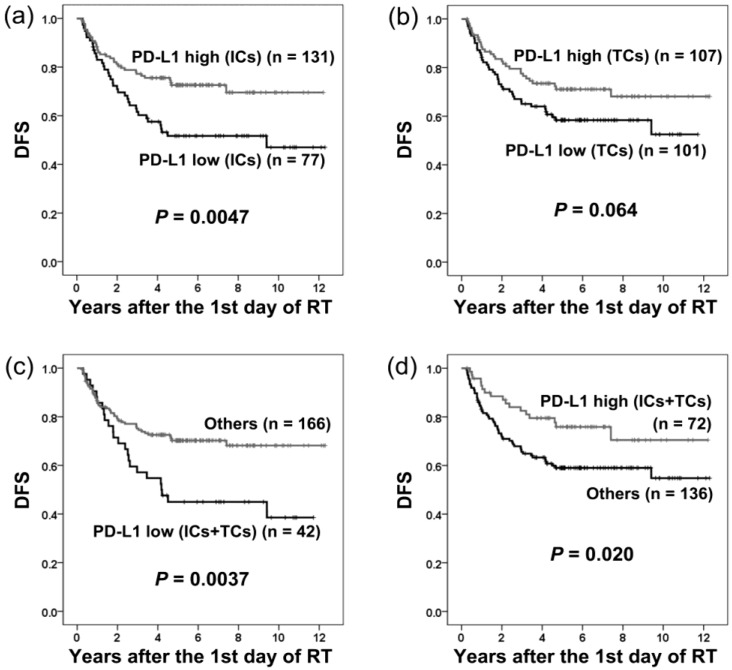
Disease-free survival (DFS) in patients with different levels of PD-L1 expression on immune cells (ICs) and tumor cells (TCs). Low PD-L1 expression on ICs (**a**) or both ICs and TCs (**c**) was associated with shorter DFS. PD-L1 expression on TCs (**b**) had no significant influence on DFS. High PD-L1 expression on both ICs and TCs (**d**) correlated with longer DFS.

The disease-free survival (DFS) of patients grouped by PD-L1 expression is shown in Figure 4. DFS was significantly shorter in patients with low PD-L1 expression on ICs (*p* = 0.0047), or both ICs and TCs (*p* = 0.0037; Figure 4).

Using univariate analysis, low PD-L1 expression on ICs (*p* = 0.0054; HR = 1.93; 95% CI = 1.21-30.7), or both ICs and TCs (*p* = 0.0045; HR = 2.04; 95% CI = 1.25–3.33), correlated with adverse prognosis for DFS. Other adverse prognostic factors included American Joint Committee on Cancer (AJCC) stage III–IV (*p* = 0.0079; HR = 2.40; 95% CI = 1.26–4.55), N2–3 (*p* = 0.013; HR = 1.84; 95% CI = 1.14–2.96), and betel quid chewing (*p* = 0.014; HR = 1.91; 95% CI = 1.14–3.21; Table 2). All other clinical parameters had no significant influence on DFS (Table 2).

Using multivariate analysis, low PD-L1 expression on ICs was an independent adverse prognostic factor (*p* = 0.0080; HR = 1.88; 95% CI = 1.18–3.00) in addition to AJCC stage III–IV (*p* = 0.0077; HR = 2.40; 95% CI = 1.26–4.58) for DFS (Table 2). Patients with betel quid chewing had a clear trend of adverse prognosis (*p* = 0.051).

### 2.5. PD-L1 Expression and Overall Survival

**Figure 5:**
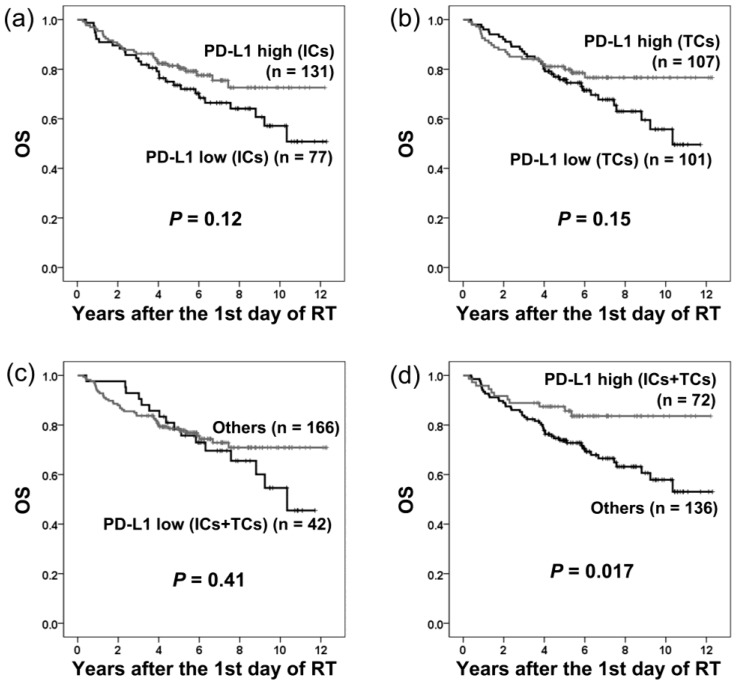
Overall survival (OS) in patients with different levels of PD-L1 expression on immune cells (ICs) and tumor cells (TCs). Low PD-L1 expression on ICs (**a**), TCs (**b**), or both ICs and TCs (**c**) had no significant influence on OS. High PD-L1 expression on both ICs and TCs (**d**) correlated with significantly longer OS.

The overall survival (OS) of patients grouped by PD-L1 expression is shown in Figure 5. OS was significantly longer in patients with high PD-L1 expression on both ICs and TCs (*p* = 0.017; Figure 5).

Using univariate analysis, high PD-L1 expression on both ICs and TCs was a favorable prognostic factor (*p* = 0.020; HR = 0.46; 95% CI = 0.24–0.88; Table 2). Significant adverse prognostic factors included Age ≥50 years (*p* = 0.033; HR = 1.78; 95% CI = 1.05–3.01), T3–4 (*p* = 0.045; HR = 1.72; 95% CI = 1.01–2.93), and N2–3 (*p* = 0.0063; HR = 2.16; 95% CI = 1.24–3.74; Table 2). All other clinical parameters had no significant influence on OS (Table 2).

Using multivariate analysis, high PD-L1 expression on both ICs and TCs was an independent favorable prognostic factor (*p* = 0.022; HR = 0.46; 95% CI = 0.24–0.89) for OS (Table 2). N2–3 (*p* = 0.0052; HR = 2.22; 95% CI = 1.27–3.89) and Age ≥50 years (*p* = 0.030; HR = 1.80; 95% CI = 1.06–3.07) were independent adverse prognostic factors for OS (Table 2).

## 3. Discussion

Previous studies on the clinical significance of PD-L1 expression in NPC showed conflicting results [8,19,20,21,22,23,24,25,26,27,28]. The EBV status could influence their results, since all previous studies either worked on a mixed cohort of EBV-positive and EBV-negative cases or did not show results of EBV-encoded small RNAs (EBER) in situ hybridization. In addition, none of these previous studies investigated the correlation between PD-L1 expression and post-radiotherapy local recurrence. Our present study is the first to report a strong association between low PD-L1 expression and post-radiotherapy local recurrence in EBV-positive NPC.

All previous studies on the clinical significance of PD-L1 expression in NPC evaluated the TCs [8,19,20,21,22,23,24,25,26,27,28], but only three of them (using clone D3, SP142 and E1L3N, respectively) also assessed the PD-L1 expression on ICs [8,19,28]. For immunohistochemistry, there are at least eight commercially available clones of PD-L1 antibodies [29]. Six of them (SP142, E1L3N, E1J2J, 28-8, 22C3 and SP263) have been shown to pass the Western blot and immunohistochemical validation, and these clones showed comparable membranous staining patterns on TCs [29]. Among these six clones, SP142 is most commonly used to evaluate PD-L1 expression on ICs. Since the percentage of PD-L1-positive cells (either ICs or TCs) can vary due to different antibody clones and immunostaining methods, finding the best cutoff value with the highest clinical significance is very important in such studies.

We found that low PD-L1 expression on ICs strongly correlated with local recurrence after radiotherapy (*p* = 0.00012; Figure 1). It was also an independent adverse prognostic factor for LRFS (*p* = 0.0062; HR = 2.74; 95% CI = 1.33–5.63) and DFS (*p* = 0.0080; HR = 1.88; 95% CI = 1.18–3.00; Table 2). In the three previous studies which also evaluated ICs for PD-L1 expression [8,19,28], they found no association between PD-L1 expression on ICs and survival, and the correlation with local recurrence was not analyzed. Although one of these studies used the same clone SP142 as ours [19], they used a different platform for immunohistochemistry (VENTANA Benchmark). In addition, their cutoff values of expression were 1% and 5%, unlike our cutoff which was defined as the median percentage of all cases. The difference in staining platforms and cutoff values could explain the different results. Interestingly, the second study using another clone, E1L3N, found a trend of favorable prognosis in DFS (*p* = 0.072) in patients with high PD-L1 expression (≥5%) on ICs [28], which is similar to our result but with less significance. Although the clone E1L3N is less commonly used to evaluate ICs than SP142 is, it has been found that SP142 and E1L3N can have comparable staining patterns on ICs of melanoma using optimized and validated immunohistochemical assays [30]. The third study using clone D3 found an association between high PD-L1 expression on ICs and longer progression-free survival (PFS) and OS only in NPC patients with higher CD8-positive tumor infiltrating lymphocytes [8]. This study worked on only 66 NPC cases, a mixture of EBV-positive (*n* = 48) and EBV-negative (*n* = 18) ones. Since EBV-positive NPC is known to have significantly more CD8-positive tumor infiltrating lymphocytes than EBV-negative NPC [24], their findings are also in line with our results.

Recently, a few studies showed that PD-L1 expression on ICs was a favorable prognostic factor in patients with other head and neck SCCs, including oral cavity, oropharyngeal, hypopharyngeal, laryngeal, and nasal cavity SCC [31,32]. This is similar to our finding that low PD-L1 expression on ICs correlated with adverse prognosis in NPC. The PD-L1 expression level on ICs could reflect the pre-treatment immune response in the tumor microenvironment, but the mechanism underlying this prognostic influence needs further investigation.

In our NPC patients, low PD-L1 expression on TCs also correlated with local recurrence after radiotherapy (*p* = 0.013; Figure 1). The association with local recurrence was even stronger in patients with low PD-L1 expression on both ICs and TCs (*p* = 0.000044; Figure 1). High PD-L1 expression on both ICs and TCs was an independent favorable prognostic factor for OS (*p* = 0.022; HR = 0.46; 95% CI = 0.24–0.89; Table 2). In seven previous studies using the antibody clone E1L3N, high PD-L1 expression on TCs was found to correlate with shorter OS [23,26,27], shorter DFS [23], shorter PFS [21,25], longer PFS [22], or longer OS and DFS [28]. One study each using clone ab58810 [24], SP263 [20], SP142 [19], and D3 [8] found no association between PD-L1 expression on TCs and survival. The difference from our results could be due to either different antibody clones [20,21,22,23,24,25,26] or different immunostaining methods and cutoff values [19]. Interestingly, two of these four studies did find an association between high PD-L1 expression on TCs and favorable prognosis in a subset of patients [8,28]. One found an association with longer PFS and OS only in NPCs with more CD8-positive lymphocytes [8], whereas the other found as association with longer OS only in NPCs with more CD3-positive lymphocytes [28]. As previously mentioned, their findings are compatible with our results, since EBV-positive NPC is known to have more abundant CD8-positive and CD3-positive tumor infiltrating lymphocytes [24].

Previous studies on the prognostic value of PD-L1 expression on TCs of other head and neck SCCs also showed inconsistent conclusions. Some studies showed no prognostic significance [31,32], whereas others found an adverse prognostic effect [33,34]. Interestingly, a recent study showed a correlation between PD-L1 expression on TCs and longer DFS and OS in patients with locally advanced oral cancer [35]. Since a large proportion of their patients received post-operative radiotherapy or chemoradiotherapy, the radiotherapy could play some role in this unique favorable prognostic effect, which is similar to our finding in NPC patients.

Despite the strong association between low PD-L1 expression and local recurrence in our patients, we found that PD-L1 expression had no influence on neck recurrence or distant metastasis. Our results suggest that the effect of PD-L1 expression was limited to the microenvironment of primary tumor site in EBV-positive NPC patients who received radiotherapy. Although concurrent chemotherapy might also play some role in the majority of our patients, the strong correlation with local recurrence, but not distant metastasis, suggests that PD-L1 expression mainly affects the outcome of local radiotherapy. For decades, most research to improve radiotherapy has focused on modulating the biological effects on cancer cells. Recently, we have gained a better understanding of how the tumor microenvironment plays pivotal roles in determining treatment outcomes [36]. Since EBV-positive NPC has a unique microenvironment rich in lymphocytes, its response to radiotherapy could differ significantly from other types of cancers. In addition, the EBV infection in cancer cells could further complicate the immune response in the tumor microenvironment [37,38]. Although the PD-1/PD-L1 axis is a potent inhibitor of immune activation, it has been suggested that PD-L1 expression might reflect the presence of antigen-induced anti-tumor immune pressure mediated by tumor-infiltrating lymphocytes [39]. PD-L1 expression can be upregulated by T-cell secretion of interferon γ, and patients with T-cell-rich tumors expressing PD-L1 appear to have better immune surveillance [40]. This might explain for our seemingly paradoxical observation that low PD-L1 expression correlates with local recurrence in our NPC patients. A previous animal study using injected cell lines of murine breast and colon cancers showed that PD-L1 expression on TCs was upregulated after irradiation [41]. Upregulation of PD-L1 expression on TCs was also found in human rectal adenocarcinoma after chemoradiotherapy [42]. However, another study showed that the PD-L1 expression on both TC and ICs of human NPC dropped after radiotherapy [19]. Further studies are needed to clarify the role of PD-1/PD-L1 axis in the unique microenvironment of NPC.

## 4. Materials and Methods

### 4.1. Patients and Samples

**Figure 6:**
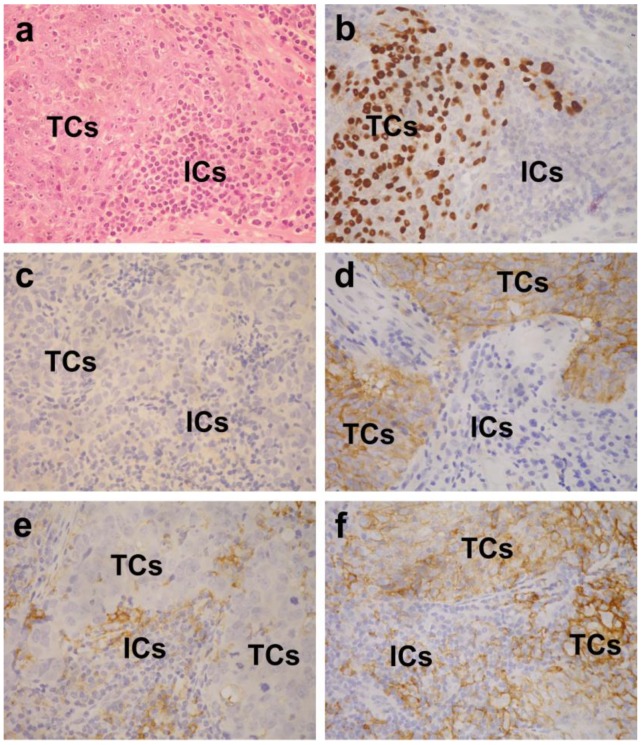
Epstein-Barr virus (EBV)-positive nasopharyngeal carcinoma (NPC) is characterized by poorly differentiated tumor cells (TCs) and many admixed immune cells (ICs) (**a**; H&E stain). Nuclear EBV-encoded small RNAs (EBER) signal is present in the TCs (**b**; in situ hybridization). Also seen are examples of cases with PD-L1-low on both TCs and ICs (**c**), PD-L1-high on TCs and PD-L1-low on ICs (**d**), PD-L1-low on TCs and PD-L1-high on ICs (**e**), and PD-L1-high on both TCs and ICs (**f**). Note that TCs have much larger nuclei than the ICs have. The original magnification of all microscopic images was × 400.

We retrospectively studied 208 EBV-positive non-keratinizing NPC patients who received intensity-modulated radiotherapy (203 of them with concurrent chemotherapy) within a period of 10 years at Chang Gung Memorial Hospital in Linkou, Taiwan. EBV positivity was defined as presence of EBV-encoded small RNAs (EBER) nuclear signals in tumor cells by in situ hybridization (Figure 6a,b). Formalin-fixed, paraffin-embedded (FFPE) tissue of primary tumor biopsy before treatment was retrieved from the Department of Pathology. The clinical information was collected by a senior radiation oncologist (N.-M. Tsang). Tumor staging was performed according to the 8th edition of American Joint Committee on Cancer (AJCC) Cancer Staging Manual [43]. This study had been approved by the Institutional Review Board of Chang Gung Memorial Hospital (IRB No. 201701694B0), Permission date (15 November 2017).

### 4.2. Evaluation of PD-L1 Expression

An immunohistochemical study was performed on 5-µm-thick sections of FFPE tumor tissue using a fully automated immunohistochemistry and in situ hybridization machine (BOND-MAX; Leica, Wetzlar, Germany). The primary antibody used was a monoclonal rabbit anti-PD-L1 antibody (clone SP142; Roche/Ventana, Tucson, AZ, USA) at a dilution of 1:100. Heat-induced epitope retrieval was performed using a citrate-based buffer (Epitope Retrieval Solution 1; Leica, Wetzlar, Germany) at 100 °C for 20 min. Under a microscope, cells with definite membranous staining were considered positive. TCs can be easily distinguished from ICs by their much larger nuclear size (Figure 6c–f). The percentages of TCs and ICs positive for PD-L1 were evaluated separately. The evaluation was first performed independently by two senior pathologists (W.-Y. Chuang and C. Hsueh) without knowing the clinical data. For discrepant results, a consensus percentage was determined by examination under a dual head microscope. For both TCs and ICs, low PD-L1 expression was defined as a percentage of positive cells lower than the median of all cases (otherwise regarded as high expression).

### 4.3. Statistical Analysis

Differences in categorical data were assessed by chi-square tests, and Yates’ correction was performed if expected frequencies less than five were encountered. The difference in age was compared with Student’s *t*-test. Survival was analyzed by the Kaplan-Meier method and compared by log-rank tests. The influence of parameters on survival was analyzed using univariate Cox regression, and parameters with significant influence were subsequently analyzed using multivariate Cox regression. A *p*-value less than 0.05 was considered statistically significant. All statistical analyses were performed using the IBM SPSS Statistics 20.0 software (SPSS Inc., Chicago, IL, USA).

## 5. Conclusions

In the present study, we evaluated the PD-L1 expression on ICs and TCs of 208 EBV-positive NPC patients. We showed for the first time that low PD-L1 expression on ICs and TCs strongly correlated with local recurrence in EBV-positive NPC patients who received radiation-based therapy. A simple immunohistochemical study for PD-L1 can be used to identify patients with a higher risk of local recurrence. These high-risk patients might benefit from more aggressive therapy in future clinical trials.

## Figures and Tables

**Table 1:** Clinical characteristics grouped by programmed death-ligand 1 (PD-L1) expression on tumor cells (TCs) or immune cells (ICs).

Characteristic	Total (*n* = 208)	PD-L1 on TCs		*p*-Value	PD-L1 on ICs		*p*-Value
		Low (*n* = 101)	High (*n* = 107)		Low (*n* = 77)	High (*n* = 131)	
Pre-treatment							
Age							
Mean ± SD	49 ± 11	50 ± 11	48 ± 11	0.22	49 ± 11	49 ± 11	0.86
Median (min; max)	49 (20; 84)	49 (25; 84)	48 (20; 73)		49 (21; 81)	49 (20; 84)	
Gender							
Male	146 (70)	70 (69)	76 (71)	0.79	53 (69)	93 (71)	0.74
Female	62 (30)	31 (31)	31 (29)		24 (31)	38 (29)	
Smoking							
Yes	109 (52)	58 (57)	51 (48)	0.16	37 (48)	72 (55)	0.33
No	99 (48)	43 (43)	56 (52)		40 (52)	59 (45)	
Alcohol							
Yes	56 (27)	29 (29)	27 (25)	0.57	23 (30)	33 (25)	0.46
No	152 (73)	72 (71)	80 (75)		54 (70)	98 (75)	
Betel quid							
Yes	40 (19)	23 (23)	17 (16)	0.21	19 (25)	21 (16)	0.13
No	168 (81)	78 (77)	90 (84)		58 (75)	110 (84)	
T category							
T1-2	104 (50)	50 (50)	54 (50)	0.89	36 (47)	68 (52)	0.47
T3-4	104 (50)	51 (50)	53 (50)		41 (53)	63 (48)	
N category							
N0-1	101 (49)	49 (49)	52 (49)	0.99	39 (51)	62 (47)	0.64
N2-3	107 (51)	52 (51)	55 (51)		38 (49)	69 (53)	
M category							
M0	205 (99)	101 (100)	104 (97)	0.27	76 (99)	129 (99)	1.0
M1	3 (1)	0 (0)	3 (3)		1 (1)	2 (1)	
AJCC Stage							
I–II	55 (26)	29 (29)	26 (24)	0.47	22 (29)	33 (25)	0.59
III–IV	153 (74)	72 (71)	81 (76)		55 (71)	98 (75)	
Treatment							
Chemotherapy							
Yes	203 (98)	97 (96)	106 (99)	0.33	75 (97)	128 (98)	1.0
No	5 (2)	4 (4)	1 (1)		2 (3)	3 (2)	
Post-treatment							
Local recurrence							
Yes	32 (15)	22 (22)	10 (9)	0.013 *	20 (26)	12 (9)	0.0012 *
No	176 (85)	79 (78)	97 (91)		57 (74)	119 (91)	
Neck recurrence							
Yes	34 (16)	21 (21)	13 (12)	0.09	15 (20)	19 (15)	0.35
No	174 (84)	80 (79)	94 (88)		62 (80)	112 (85)	
Distant metastasis							
Yes	35 (17)	20 (20)	15 (14)	0.27	18 (23)	17 (13)	0.053
No	173 (83)	81 (80)	92 (86)		59 (77)	114 (87)	

SD, standard deviation; AJCC, American Joint Committee on Cancer; * *p* < 0.05.

**Table 2:** Association between prognostic factors and survival.

Factor				Hazard Ratio	95% CI	*p*-Value
Local recurrence-free survival						
	Univariate analysis					
		PD-L1 (ICs-low vs. ICs-high)		2.99	1.46–6.12	0.0028 *
		PD-L1 (TCs-low vs. TCs-high)		2.34	1.11–4.95	0.026 *
			PD-L1 (ICs/TCs both low vs. others)	3.48	1.74–6.98	0.00045 *
			PD-L1 (ICs/TCs both high vs. others)	0.33	0.13–0.86	0.023 *
		Age (≥50 years vs. <50 years)		1.28	0.64–2.57	0.48
		Gender (male vs. female)		0.54	0.27–1.09	0.085
		Smoking (yes vs. no)		0.70	0.35–1.40	0.31
		Alcohol drinking (yes vs. no)		0.87	0.39–1.93	0.73
		Betel quid chewing (yes vs. no)		1.00	0.41–2.42	1.00
		AJCC stage (III–IV vs. I–II)		1.78	0.73–4.32	0.21
			T category (T3–4 vs. T1–2)	1.60	0.79–3.23	0.20
			N category (N2–3 vs. N0–1)	1.53	0.76–3.10	0.24
			M category (M1 vs. M0)	0.05	0–2.0 × 10^5^	0.70
		Chemotherapy (yes vs. no)		0.40	0.10–1.66	0.21
	Multivariate analysis					
		PD-L1 (ICs-low vs. ICs-high)		2.74	1.33–5.63	0.0062 *
		PD-L1 (TCs-low vs. TCs-high)		2.07	0.98–4.40	0.058
Disease-free survival						
	Univariate analysis					
		PD-L1 (ICs-low vs. ICs-high)		1.93	1.21–3.07	0.0054 *
		PD-L1 (TCs-low vs. TCs-high)		1.55	0.97–2.48	0.067
			PD-L1 (ICs/TCs both low vs. others)	2.04	1.25–3.33	0.0045 *
			PD-L1 (ICs/TCs both high vs. others)	0.53	0.31–0.91	0.022 *
		Age (≥50 years vs. <50 years)		0.99	0.62–1.58	0.97
		Gender (male vs. female)		0.77	0.48–1.25	0.29
		Smoking (yes vs. no)		0.78	0.49–1.24	0.29
		Alcohol drinking (yes vs. no)		1.40	0.86–2.29	0.18
		Betel quid chewing (yes vs. no)		1.91	1.14–3.21	0.014 *
		AJCC stage (III–IV vs. I–II)		2.40	1.26–4.55	0.0079 *
			T category (T3–4 vs. T1–2)	1.60	1.00–2.56	0.050
			N category (N2–3 vs. N0–1)	1.84	1.14–2.96	0.013 *
			M category (M1 vs. M0)	0.05	0–617	0.53
		Chemotherapy (yes vs. no)		0.96	0.24–3.91	0.95
	Multivariate analysis					
		PD-L1 (ICs-low vs. ICs-high)		1.88	1.18–3.00	0.0080 *
		Betel quid chewing (yes vs. no)		1.68	0.99–2.83	0.051
		AJCC stage (III–IV vs. I–II)		2.40	1.26–4.58	0.0077 *
Overall survival						
	Univariate analysis					
		PD-L1 (ICs-low vs. ICs-high)		1.51	0.90–2.55	0.12
		PD-L1 (TCs-low vs. TCs-high)		1.48	0.87–2.51	0.15
			PD-L1 (ICs/TCs both low vs. others)	1.28	0.72–2.28	0.41
			PD-L1 (ICs/TCs both high vs. others)	0.46	0.24–0.88	0.020 *
		Age (≥50 years vs. <50 years)		1.78	1.05–3.01	0.033 *
		Gender (male vs. female)		0.71	0.42–1.22	0.22
		Smoking (yes vs. no)		0.87	0.52–1.47	0.60
		Alcohol drinking (yes vs. no)		0.88	0.49–1.59	0.68
		Betel quid chewing (yes vs. no)		1.19	0.63–2.25	0.60
		AJCC stage (III–IV vs. I–II)		1.83	0.95–3.54	0.073
			T category (T3–4 vs. T1–2)	1.72	1.01–2.93	0.045 *
			N category (N2–3 vs. N0–1)	2.16	1.24–3.74	0.0063 *
			M category (M1 vs. M0)	2.41	0.33–17.5	0.39
		Chemotherapy (yes vs. no)		0.77	0.19–3.14	0.71
	Multivariate analysis					
		PD-L1 (ICs/TCs both high vs. others)		0.46	0.24–0.89	0.022 *
		Age (≥50 years vs. <50 years)		1.80	1.06–3.07	0.030 *
		T category (T3–4 vs. T1–2)		1.53	0.89–2.62	0.12
		N category (N2–3 vs. N0–1)		2.22	1.27–3.89	0.0052 *

95% CI, 95% confidence interval; ICs, immune cells; TCs, tumor cells; AJCC, American Joint Committee on Cancer; * *p* < 0.05.
